# Balanced trafficking between the ER and the Golgi apparatus increases protein secretion in yeast

**DOI:** 10.1186/s13568-018-0571-x

**Published:** 2018-03-12

**Authors:** Jichen Bao, Mingtao Huang, Dina Petranovic, Jens Nielsen

**Affiliations:** 1 Department of Biology and Biological Engineering, Chalmers University of Technology, 41296 Gothenburg, Sweden; 2 Novo Nordisk Foundation Center for Biosustainability, Chalmers University of Technology, 41296 Gothenburg, Sweden; 3 Novo Nordisk Foundation Center for Biosustainability, Technical University of Denmark, 2970 Hørsholm, Denmark

**Keywords:** Retrograde trafficking, COPI vesicle, Protein secretion, *GLO3*, *Saccharomyces cerevisiae*

## Abstract

The yeast *Saccharomyces cerevisiae* is widely used as a cell factory to produce recombinant proteins. However, *S. cerevisiae* naturally secretes only a few proteins, such as invertase and the mating alpha factor, and its secretory capacity is limited. It has been reported that engineering protein anterograde trafficking from the endoplasmic reticulum to the Golgi apparatus by the moderate overexpression of *SEC16* could increase recombinant protein secretion in *S. cerevisiae*. In this study, the retrograde trafficking in a strain with moderate overexpression of *SEC16* was engineered by overexpression of ADP-ribosylation factor GTP activating proteins, Gcs1p and Glo3p, which are involved in the process of COPI-coated vesicle formation. Engineering the retrograde trafficking increased the secretion of α-amylase but did not induce production of reactive oxygen species. An expanded ER membrane was detected in both the *GCS1* and *GLO3* overexpression strains. Physiological characterizations during batch fermentation showed that *GLO3* overexpression had better effect on recombinant protein secretion than *GCS1* overexpression. Additionally, the *GLO3* overexpression strain had higher secretion of two other recombinant proteins, endoglucanase I from *Trichoderma reesei* and glucan-1,4-α-glucosidase from *Rhizopus oryzae*, indicating overexpression of *GLO3* in a *SEC16* moderate overexpression strain might be a general strategy for improving production of secreted proteins by yeast.

**Electronic supplementary material:**

The online version of this article (10.1186/s13568-018-0571-x) contains supplementary material, which is available to authorized users.

## Introduction

The yeast *Saccharomyces cerevisiae* is a well-known eukaryal cell factory for producing many valuable chemicals and proteins (Huang et al. 2014), due to its fast growth and robustness (Hong and Nielsen 2012) and being Generally Recognized As Safe (GRAS) (Shusta et al. 1998). Furthermore, the ease of doing genetic manipulation, the availability of many molecular tools, datasets and databases facilitate work with *S. cerevisiae* (Cox and Mann 2011; Hawkins et al. 2010; Reaves and Rabinowitz 2011; Snyder and Gallagher 2009). Like other *Eukarya*, *S. cerevisiae* has a secretory pathway, which means that recombinant proteins can undergo folding, disulfide bond formation, glycosylation, and be transported out of the cell (Hou et al. 2012b). However, the secretory capacity of wild type *S. cerevisiae* is limited (Idiris et al. 2010), and improving the secretory capacity through engineering of this pathway would reduce the cost of downstream processes (Nielsen 2013). Over the past few years, we have successfully improved recombinant protein secretion by *S. cerevisiae* (Hou et al. 2012a; Huang et al. 2015; Liu et al. 2012; Martínez et al. 2016). Liu et al. reported that different signal peptides have different effects on the secretion of recombinant proteins (Liu et al. 2012). The signal peptide is recognized by the signal recognition particle on the endoplasmic reticulum (ER), and the recombinant protein is co-translationally translocated into the ER (Plath et al. 1998). The nascent peptide undergoes disulfide bond formation in the ER with the assistance of the chaperone Pdi1p. Schröder and Robinson reported that overexpressing *PDI1* improves recombinant protein secretion in yeast (Robinson et al. 1994; Schröder 2008). High level of recombinant protein expression may, however, cause ER stress due to protein misfolding. When unfolded proteins accumulate in the ER, the so-called unfolded protein response (UPR) will be triggered to relieve ER stress (Hou et al. 2012b). Overexpression of the transcription factor Hac1p, which is one of the key regulators of the UPR, enhances the secretion of α-amylase (Valkonen et al. 2003). Additionally, engineering the vesicle trafficking successfully increases heterologous protein secretion. Hou et al. found that overexpression of Sly1p, an SM (Sec1/Munc-18) family protein that regulates trafficking from the ER to the Golgi, enhances α-amylase secretion but not for human insulin precursor or invertase, whereas overexpression of Sec1p, which also belongs to the SM family, improves the secretion of all three proteins (Hou et al. 2012a). Recently, we found that engineering the anterograde trafficking between the ER to the Golgi by moderate overexpression of *SEC16* increases recombinant protein secretion (Bao et al. 2017). Sec16p, an ER peripheral protein, accumulates at the ER exit sites (ERESs) and serves as a scaffold for the formation of coat protein complex II (COPII) vesicles which transport the cargo protein from the ER to the Golgi (Jensen and Schekman 2011; Supek et al. 2002). In *S. cerevisiae*, the native level of Sec16p is lower than that of the other COPII vesicle proteins, which may cause a limitation in the COPII vesicle formation (Feizi et al. 2013). Moderate expression of *SEC16* generates more ERESs facilitating the formation of COPII vesicles. A less expanded ER combined with the result of reactive oxygen species (ROS) staining indicates a reduced level of ER stress in *SEC16* overexpression strain (Bao et al. 2017). The early secretory pathway is bidirectional: anterograde trafficking mediates the transportation of cargo protein from the ER to the Golgi, and the retrograde trafficking mediated by coat protein complex I (COPI) vesicles retrieves the necessary components for continued anterograde trafficking (Pelham 1995; Poon et al. 1999). The process of COPI-coated vesicle formation is similar to that of COPII-coated vesicles. GDP bounded Arf1p is activated by guanine exchange factor (ArfGEF) by exchanging GDP with GTP, which triggers the formation of COPI-coated vesicles (Poon et al. 1999). Following this the coatomers are recruited by the activated Arf1p-GTP followed by recruitment of the cargo proteins. The COPI-coated vesicles are formed subsequently. The mature COPI vesicles are prompted by the disassembly of coatomers, which is caused by GTP hydrolysis via the GTP activating proteins (GAPs) Gcs1p and Glo3p (Poon et al. 1999).

In our previous study, we found that moderate overexpression of *SEC16* increases secretion of a range of heterologous proteins by *S. cerevisiae* (Bao et al. 2017). Overexpression of *SEC16* provides more ERESs for cargo protein export. However, the enhanced flux of ER-to-Golgi transportation may bring excess lipids and ER associated proteins, such as v-SNARE proteins, to the Golgi via COPII vesicles (Szul and Sztul 2011). Sequential coupling between COPII and COPI vesicles is important to coordinate and direct bi-directional vesicular trafficking between the ER and the Golgi apparatus (Aridor et al. 1995), suggesting that improving the recycling of these components would further increase the trafficking flow to enhance protein secretion.

Here we used the recombinant protein α-amylase from *Aspergillus oryzae* as a reporter to evaluate the secretory capacity in yeast. We amplified the retrograde trafficking pathway by overexpressing GAPs, Gcs1p and Glo3p, in a *SEC16*-overexpression strain to further increase the secretion of heterologous proteins in yeast.

## Materials and methods

### Strains and media

The strains and plasmids used in this study are listed in Table 1. The primers used in this study are listed in Additional file 1: Table S1. Yeast strain CEN.PK530-6CK (*MATa URA3 HIS3 LEU2 TRP1 SUC2 MAL2*-*8*^*c*^ *tpi1(41*-*707)::loxP P*_*GPD*_-*SEC16*) was used as a heterologous protein secretion host. Plasmid pAlphaAmyCPOT, which contains α-amylase from *Aspergillus oryzae* with α-factor leader was transformed into CEN.PK530-6CK, named YIGS16 (Bao et al. 2017). The yeast strains CEN.PK530-6CK + EG and CEN.PK530-6CK + AGL were constructed in the same way as YIGS16, that is the plasmids pAlphaTrEGCPOT and pCP-aGLA were transformed into CEN.PK530-6CK respectively, resulting CEN.PK530-6CK + EG and CEN.PK530-6CK + AGL (Bao et al. 2017).

**Table 1:** Plasmids and strains used in this study

Name	Description	References
Plasmids		
CPOTud	TPI promoter and terminator from *S. cerevisiae*, *POT* marker from *Schizosaccharomyces pombe* (2 μ)	Liu et al. (2012)
pAlphaAmyCPOT	α factor leader with α-amylase gene inserted into CPOTud	Liu et al. (2012)
pAlphaTrEGCPOT	α factor leader with endoglucanase I gene inserted into CPOTud	Bao et al. (2017)
pCP-aGLA	α factor leader with glucan 1,4-α-glucosidase gene inserted into CPOTud	Huang et al. (2015)
Strains		
CEN.PK530-6CK	*MATa URA3 HIS3 LEU2 TRP1 SUC2 MAL2*-*8*^*c*^ *tpi1(41*-*707)::loxP P*_*GPD*_-*SEC16*	Bao et al. (2017)
YIGS16	CEN.PK530-6CK with pAlphaAmyCPOT	Bao et al. (2017)
YIGCS1	YIGS16 *amdSYM P*_*TEF*_-*GCS1*	This study
YIGLO3	YIGS16 *amdSYM P*_*TEF*_-*GLO3*	This study
AACK	*MATa URA3 HIS3 LEU2 TRP1 SUC2 MAL2*-*8*^*c*^ *tpi1(41*-*707)::loxP* with pAlphaAmyCPOT	Bao et al. (2017)
AACK-GCS1	AACK *amdSYM P*_*TEF*_-*GCS1*	This study
AACK-GLO3	AACK *amdSYM P*_*TEF*_-*GLO3*	This study
YIGLO3GCS1	YIGS16 *P*_*TEF*_-*GLO3 amdSYM P*_*TEF*_-*GCS1*	This study
CEN.PK530-6CK + EG	CEN.PK530-6CK with pAlphaTrEGCPOT	This study
CEN.PK530-6CK + AGL	CEN.PK530-6CK with pCP-aGLA	This study
GLO3 + EG	CEN.PK530-6CK *amdSYM P*_*TEF*_-*GLO3* with pAlphaTrEGCPOT	This study
GLO3 + AGL	CEN.PK530-6CK *amdSYM P*_*TEF*_-*GLO3* with pCP-aGLA	This study

The promoter replacement cassette “UP(GCS1)-amdSYM(GCS1)-TEF(GCS1)-DW(GCS1)” for *GCS1* was constructed by the following steps: The “UP(GCS1)” fragment is the upstream flanking region, which was amplified from the yeast genome by using primers GCS1-up-100-F and GCS1-up-100-R-amds; the “amdSYM(GCS1)” is the selection marker, which was amplified from plasmid pamdSYM by primers amdS-F and REC-amdSYM-R(gcs1); The “TEF(GCS1)” fragment is the promoter *P*_*TEF*_ which was amplified by REC-P-TEF1-F(GCS1) and gcs1-TEF1-R based on the yeast genome; The “DW(GCS1)” fragment is the downstream flanking region, which was amplified from the yeast genome by primers GCS1-F and GCS1-MID-R; then the four fragments “UP(GCS1)”, “amdSYM”, “TEF” and “DW(GCS1)” were jointed together by fusion PCR, resulting in the replacement cassette “UP(GCS1)-amdSYM(GCS1)-TEF(GCS1)-DW(GCS1)”. Similarly, the promoter replacement cassette “UP(GLO3)-amdSYM(GLO3)-TEF(GLO3)-DW(GLO3)” was constructed by using primer pairs “GLO3-up-120-F and GLO3-up-120-R-amds”, “amdS-F and REC-amdSYM-R(glo3)”, “REC-P-TEF1-F(glo3) and glo3-TEF1-R” and “GLO3-F and GLO3-MID-R” for amplification of fragments “UP(GLO3)”, “amdSYM(GLO3)”, “TEF(GLO3)” and “DW(GLO3)” respectively, and then jointing together by fusion PCR. The replacement of the *GCS1* native promoter or the GLO3 native promoter by the TEF promoter was completed by transforming the replacement cassette “UP(GCS1)-amdSYM(GCS1)-TEF(GCS1)-DW(GCS1)” or “UP(GLO3)-amdSYM(GLO3)-TEF(GLO3)-DW(GLO3)” into the cell. Yeast strain AACK-GCS1 was constructed by transformation of the “UP(GCS1)-amdSYM(GCS1)-TEF(GCS1)-DW(GCS1)” cassette to strain AACK. The strains AACK-GLO3, YIGLO3, GLO3 + EG and GLO3 + AGL were constructed by transformation of the “UP(GLO3)-amdSYM(GLO3)-TEF(GLO3)-DW(GLO3)” cassette to strains AACK, YIGS16, CEN.PK530-6CK + EG and CEN.PK530-6CK + AGL, respectively. The transformants were selected on acetamide plate (Solis-Escalante et al. 2013). The acetamide medium contained 3 g/L KH_2_PO_4_, 0.5 g/L MgSO_4_·7H_2_O, 0.6 g/L acetamide, 6.6 g/L K_2_SO_4_, 1 mL/L of a trace element solution (Verduyn et al. 1992) and 1 mL/L a vitamin solution (Verduyn et al. 1992). The yeast strain YIGLO3GCS1 was constructed by the following steps: The amdSYM marker was removed from YIGLO3 genome in YPD medium, and the colonies were counter-selected on a fluoroacetamide plate as described (Solis-Escalante et al. 2013). Then the promoter replacement cassette “UP(GCS1)-amdSYM(GCS1)-TEF(GCS1)-DW(GCS1)” was transformed into the YIGLO3 amdSYM-removal strain, resulting in YIGLO3GCS1.

Under non-selective conditions, yeast strains were grown in YPD medium containing 10 g/L yeast extract, 20 g/L peptone and 20 g/L glucose. The yeast strains were cultured in SD-2xSCAA (Wittrup and Benig 1994) medium for recombinant protein production containing 20 g/L glucose, 6.9 g/L yeast nitrogen base without amino acids, 190 mg/L arginine, 400 mg/L aspartic acid, 1260 mg/L glutamic acid, 130 mg/L glycine, 140 mg/L histidine, 290 mg/L isoleucine, 400 mg/L leucine, 440 mg/L lysine, 108 mg/L methionine, 200 mg/L phenylalanine, 220 mg/L threonine, 40 mg/L tryptophan, 52 mg/L tyrosine, 380 mg/L valine, 1 g/L BSA, 5.4 g/L Na_2_HPO4, and 8.56 g/L NaH_2_PO_4_·H_2_O (pH = 6.0 by NaOH). The cultivation time for tube fermentation was 96 h. In the bioreactor batch fermentations, SD-2xSCAA medium was used. Strains were inoculated into 600 mL of SD-2xSCAA medium in a 1-L bioreactor (DasGip, Jülich, Germany) at 30 °C. The bioreactor system was run at 600 rpm, and 36 L/h air flow, and the pH value was maintained at 6 by the addition of NaOH.

### Analytical methods

The cell dry weight and the concentration of glucose, ethanol and glycerol were detected as described previously (Hou et al. 2012a). Briefly, a high performance liquid chromatography (HPLC) (Dionex, Sunnyvale, CA, USA) was used with an Aminex HPX-87H column (Bio-Rad, Hercules, USA) at 65 °C. 5 mM H_2_SO_4_ was used as mobile phase with a flow rate of 0.6 mL/min.

### Enzyme activity quantification

The amylase enzyme activity was quantified by the assay kit (Megazyme K-CERA, Wicklow, Ireland). α-Amylase from *A. oryzae* was used as a standard. The enzyme activity of endoglucanase was measured by a cellulase assay kit (Megazyme K-CELLG3) at 50 °C for 10 min. The enzyme activity of glucan 1,4-α-glucosidase was measured by an amyloglucosidase assay kit (Megazyme R-AMGR3, Wicklow, Ireland).

### Intracellular α-amylase extraction

Yeast cells were harvested and washed by 1× phosphate buffer solution (pH 7.4) (PBS) twice. Then the cell pellets were resuspended in 1 mL 1× PBS with 10 μL of halt protease inhibitor cocktail (Thermo Fisher, Waltham, MA, USA). Next, 500 μL cell solution was transferred into 1.0 mm silica spheres lysing matrix tube (MP Biomedicals, Santa Ana, CA, USA). The cells were lysed using fastprep-24 tissue and cell homogenizer (MP Biomedicals, Santa Ana, CA, USA) at 6.5 m/s for 2 min. The cells were kept on ice during the 5 min interval between the two runs. The supernatant was collected by centrifugation for α-amylase quantification. The cell wall associated α-amylase was considered as intracellular α-amylase.

### Reactive oxygen species measurement

The cells for reactive oxygen species (ROS) measurement were cultivated in tube at 30 °C and collected when OD_600_ reached 1. Then 1 OD_600_ of the cells of each samples were washed by 1× PBS twice and 50 mM sodium citrate buffer (pH 5) (SCB) once. The cell pellets were resuspended in 1 mL of SCB with 1 μL of 50 mM dihydrorhodamine 123 (Thermo Fisher, Waltham, MA, USA), and then were incubated in the dark at room temperature for 30 min. After the incubation, the cells were washed and resuspended in 1 mL of SCB. 200 μL of the cell samples were loaded into 96-well black plate for detection by fluorescence microplate reader (FLUOstar Omega, BMG LABTECH, Germany) with a 485-nm excitation filter and a 520-nm emission filter.

### ER membrane staining

The cells for ER membrane staining were cultivated in tube at 30 °C and collected when OD_600_ reached 1. Then 1 OD_600_ of the cells were washed by 1× PBS twice and Hanks’ Balance Salt Solution without phenol red (HBSS) once. The cell pellets were resuspended in 1 mL of HBSS with 5 μL of ER-Tracker Blue-White DPX (Thermo Fisher, Waltham, MA, USA), and incubated for 30 min at 30 °C. After the incubation, the cells were washed and resuspended in 1 mL of HBSS. 200 μL of the cell samples were loaded into 96-well black plate for detection by fluorescence microplate reader with a 355-nm excitation filter and a 590-nm emission filter.

### Statistical method

Student’s t test was used for statistical significance testing in this study.

## Results

### Overexpression of *GCS1* or *GLO3* improves the titer of α-amylase in the *SEC16*-overexpression strain

**Fig. 1:**
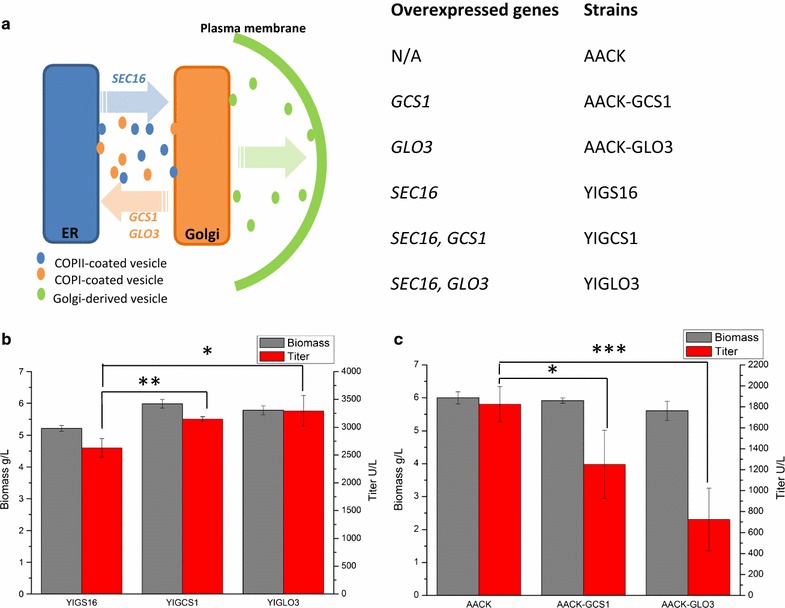
Overexpression of *GCS1* or *GLO3* by promoter replacement in the *SEC16*-overexpression strain (YIGS16) has a positive effect on α-amylase secretion. **a** Constructions of the vesicle trafficking engineering strains. *SEC16* is involved in COPII vesicle formation; *GCS1* and *GLO3* are involved in COPI vesicle formation. **b** The titer and biomass of YIGS16, YIGCS1 and YIGLO3. **c** The titer and biomass of AACK, AACK-GCS1 and AACK-GLO3. *P < 0.05; **P < 0.01; ***P < 0.001. Measurements are reported as the average value ± standard deviation from independent triplicates

To increase the protein secretory capacity in a *SEC16* overexpression strain by enlarging the retrograde trafficking pathway, two ARF GAPs mediating COPI vesicle formation, Gcs1p and Glo3p were overexpressed (Fig. 1a). Overexpression of *GCS1* or *GLO3* enhanced the titer of α-amylase 19.7 and 25.1%, respectively, when the cells were grown in tube cultures for 96 h (Fig. 1b). The final biomass yield of the *GCS1* or *GLO3* overexpression strains, YIGCS1 and YIGLO3, were slightly higher than the reference strain YIGS16 (Fig. 1b). Interestingly, we found that overexpression of *GCS1* or *GLO3* in the reference strain AACK (yeast strain without overexpression of *SEC16*) significantly decreased the titer of α-amylase secretion (Fig. 1c). We also tried to overexpress *GCS1* and *GLO3* simultaneously in YIGS16, yet the α-amylase titer did not further increase compared with that of single overexpression of *GLO3* in YIGS16 (resulting in strain YIGLO3) (Additional file 1: Figure S1). This may be due to a redundant function provided by *GCS1* and *GLO3,* each one ensuring sufficient vesicular transport from the Golgi apparatus to the ER (Poon et al. 1999). Hence, only overexpression of either *GCS1* or *GLO3* was considered in subsequent experiments.

### Overexpression of *GCS1* or *GLO3* does not increase ROS

Recombinant protein production may cause ROS accumulation in cells due to the requirement for additional capacity requirement for oxidative protein folding in the ER (Tu and Weissman 2002; Tyo et al. 2012). In this study, we aimed to return more necessary components for the anterograde trafficking process in YIGS16 to strengthen the recombinant protein transportation by amplifying the retrograde trafficking, and we therefore evaluated how the ROS level was altered in the strains with a more balanced vesicle trafficking. From this analysis we found that even though the *GCS1* and *GLO3* overexpression strains had increased α-amylase secretion this was not associated with additional ROS accumulation (Additional file 1: Figure S2). This implied that overexpression of *GCS1* or *GLO3* did not cause increased cellular stress.

### Engineering the retrograde trafficking increases the surface of the ER membrane

**Fig. 2:**
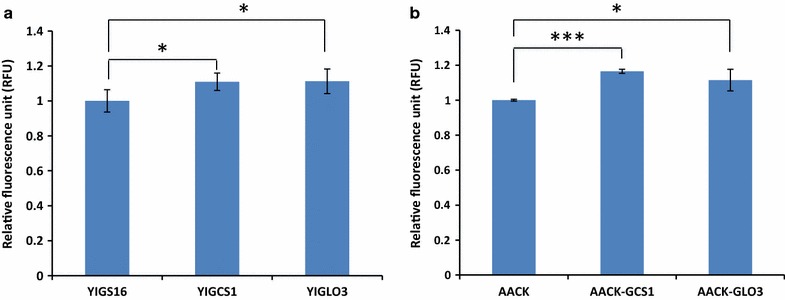
The amounts of ER membrane of strains **a** YIGS16, YIGCS1 and YIGLO3. **b** AACK, AACK-GCS1 and AACK-GLO3 using ER-Tracker Blue-White DPX. The ER membrane changes were quantified by their fluorescence intensity. *P < 0.05; ***P < 0.001. Measurements are reported as the average value ± standard deviation from independent triplicates

As one of the functions of the retrograde transportation is retrieving the necessary proteins with the lipids for the continuous anterograde trafficking (Poon et al. 1999), we were interested to test if there was any change in ER membrane surface in the engineered strains. We have shown previously that moderate expression of *SEC16* reduced ER membrane surface (AACK) so we therefore measured the ER membrane surfaces in YIGS16, YIGCS1 and YIGLO3 by ER labeling with the ER-Tracker Blue-white DPX (Echevarria et al. 2003), and found that the surfaces of ER membranes in YIGCS1 and YIGLO3 were slightly higher than in YIGS16 (Fig. 2a). Meanwhile, we also stained the ER membranes of AACK, AACK-GCS1 and AACK-GLO3, and *GCS1* and *GLO3* overexpression in AACK also resulted in an increase in ER surface area (Fig. 2b). This suggested that the ER membrane was enlarged when *GCS1* or *GLO3* were overexpressed. Enlarged ER membrane was likely a result of more lipids returned to the ER by enhanced retrograde trafficking.

### Strain YIGLO3 has higher α-amylase yield throughout the cultivation

**Fig. 3:**
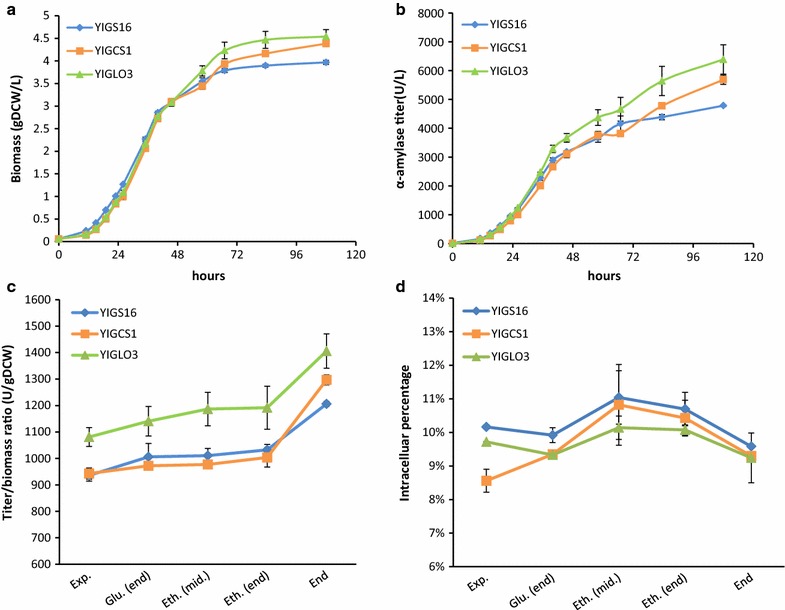
Batch fermentation of the strain YIGS16, YIGCS1 and YIGLO3. The time course of **a** the biomass and **b** the α-amylase titer. **c** The titer/biomass ratio and **d** the percentage of intracellular α-amylase of the three strains at five different time points. Measurements are reported as the average value ± standard deviation from independent triplicates

As both strains YIGCS1 and YIGLO3 showed increased final α-amylase titer in tube fermentations (Fig. 1b), we investigated α-amylase secretion throughout a fermentation process. Strains were therefore cultured in batch bioreactors, and samples were taken for analysis throughout the fermentation (Fig. 3a, b). The secreted α-amylase yield (titer/biomass ratio) was calculated at five stages of the process, which included the exponential growth phase (OD_600nm_ ≈ 1), the end of the glucose growth phase, the middle of the ethanol growth phase, the end of the ethanol growth phase and at the end of the fermentation. The α-amylase yield was higher for YIGLO3 in all stages compared with the reference strain YIGS16, while YIGCS1 only showed a higher α-amylase yield in the end (Fig. 3c). There was no significant difference in the percentage of intracellular α-amylase and intracellular α-amylase per cell for the three strains (Fig. 3d and Additional file 1: Figure S3).

Besides increased α-amylase secretion, physiological changes were also found for the engineered strains, especially for YIGLO3. Both YIGCS1 and YIGLO3 had higher maximum specific growth rates and higher final biomass titers compared with strain YIGS16 (Table 2, Fig. 3a). YIGLO3 had a significantly higher specific α-amylase production rate and yield of α-amylase in the glucose phase (Table 2). Additionally, YIGLO3 had a higher specific ethanol production rate, higher ethanol production peak, lower specific glycerol production rate and lower glycerol production peak compared with YIGS16 (Table 2, Additional file 1: Figures S4b, c). In contrast, no significant difference was found in these parameters between YIGCS1 and YIGS16. Furthermore, the glucose consumption profile was similar for all three strains (Additional file 1: Figure S4a), and there were no significant changes in the specific glucose uptake rates among the three strains (Table 2).

**Table 2:** Physiological parameters of YIGS16, YIGCS1 and YIGLO3

Strain	μ_max_	r_S_	r_p_	*Y*_*Sα*_	r_E_	r_G_
YIGS16	0.194 ± 0.003	1.21 ± 0.01	158.12 ± 7.80	130.47 ± 4.98	0.147 ± 0.002	0.253 ± 0.005
YIGCS1	0.226 ± 0.004***	1.16 ± 0.01**	154.36 ± 1.74	128.93 ± 2.48	0.147 ± 0.005	0.261 ± 0.007
YIGLO3	0.217 ± 0.002**	1.25 ± 0.03	182.90 ± 7.31*	145.92 ± 5.76*	0.165 ± 0.001***	0.231 ± 0.006*

*μ*_*max*_ maximum specific growth rate (h^−1^) on glucose, *r*_*S*_ specific glucose uptake rate (g/(g-DCW)/h), *r*_*P*_ specific α-amylase production rate (U/(g-DCW)/h) on glucose, *Y*_*sα*_ yield of α-amylase from glucose (U/g), *r*_*E*_ specific ethanol production rate (g/(g-DCW)/h), *r*_*G*_ specific glycerol production rate (g/(g-DCW)/h)

“*” represents the statistical significance of the difference between the parameters of YIGCS1 or YIGLO3 and those of YIGS16. *P < 0.05; **P < 0.01; ***P < 0.001. Measurements are reported as the average value ± standard deviation from independent triplicates

### Overexpression of *GLO3* improves secretion of two other recombinant proteins

**Fig. 4:**
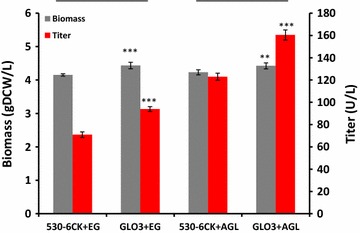
Secretion of two recombinant proteins, endoglucanase and glucan 1,4-α-glucosidase, in the reference strain and *GLO3*-overexpression strain. ***P < 0.001 Measurements are reported as the average value ± standard deviation from independent quadruplicates

To investigate whether overexpression of *GLO3* can also increase the production of other recombinant proteins, two heterologous proteins, *Trichoderma reesei* endoglucanase I and *Rhizopus oryzae* glucan-1,4-α-glucosidase were evaluated. The production of both proteins significantly increased, about 30%, in the *GLO3* overexpression strain, and just as for YIGLO3 the final biomass titer of both strains increased about 5% compared to that of the corresponding reference strain (Fig. 4).

## Discussion

ER-to-Golgi translocation of recombinant proteins is mediated by COPII-coated vesicles (Jensen and Schekman 2011). Previously, we showed that moderate overexpression (but not high-level overexpression) of *SEC16* in yeast improved protein secretion by enhancing the anterograde transport from the ER to the Golgi apparatus (Bao et al. 2017). Meanwhile, the enhanced ER-to-Golgi flux caused by expressing *SEC16* resulted in more ER membranes and ER membrane proteins to be directed to the anterograde vesicles to the Golgi, and this resulted in reduced ER membranes (Bao et al. 2017). COPI-coated vesicles are responsible for retrieving these essential components from the Golgi apparatus to the ER for continuous anterograde trafficking (Poon et al. 1999). Here by overexpression of *GCS1* or *GLO3* we increased the retrograde trafficking from the Golgi to the ER in the YIGS16 background strain expressing *SEC16* and we also detected increased amounts of ER membranes in YIGCS1 and YIGLO3. Hereby more essential components were recycled to the ER and could be reused for anterograde trafficking, resulting in increased protein secretion.

In the mammalian cells, interference of COPI vesicle formation by the addition of ArfGEF inhibitor brefeldin A shows a negative effect on ER export (Ward et al. 2001), which may result from the shortage of the protein components required for the COPII vesicle assembly that occurs since the retrograde trafficking from the Golgi to the ER was inadequate (Brandizzi and Barlowe 2013). A similar phenomenon was observed that the secretory pathway was impaired when the anterograde trafficking was disturbed by the disruption of COPII vesicle formation using Sar1p [H79G], a GTP locked version (Aridor et al. 1995; Pepperkok et al. 1998; Ward et al. 2001). These results imply that the balance between anterograde and retrograde trafficking plays an important role in the secretory pathway. Wildtype *S. cerevisiae* has a limited protein secretory capacity and secretes naturally only a few proteins such as α-factor, invertase, and aspartyl protease (Carlson et al. 1983; Idiris et al. 2010; Komano et al. 1999). In our previous study, we found that increasing the anterograde trafficking by moderate expression of *SEC16* improves the yeast secretory pathway (Bao et al. 2017), which implies the natural anterograde trafficking flow in *S. cerevisiae* is inadequate. When only *GLO3* or *GCS1* was overexpressed in the wildtype strain AACK, the titer of α-amylase was decreased. This may be due to the inadequacy of the anterograde trafficking as the retrograde trafficking was overflown. However, when *GLO3* or *GCS1* were overexpressed in the anterograde trafficking amplifying strain YIGS16, the secretory capacity could be further increased. This might be the trafficking flow between the ER and the Golgi apparatus was improved by engineering the retrograde trafficking, reaching a better balance. In the controlled condition, the effect of *GCS1* overexpression on protein secretion was inconspicuous compared with *GLO3*. This is due to that Glo3p plays a major role in the COPI vesicle formation (Poon et al. 1999), which could also explain AACK-GLO3 has an even lower α-amylase titer than AACK-GCS1 (Fig. 1c).

ER expansion by the deletion of the lipid-regulator *OPI1* alleviates ER stress and increases the secretion of IgG in *S. cerevisiae* (de Ruijter et al. 2016; Schuck et al. 2009). Interestingly, the overexpression of *GCS1* or *GLO3* increases the amounts of ER membranes in AACK and YIGS16, but has different effects on the secretion, which might be due to the two different manners of ER expansion. In our case, the ER was enlarged by amplifying the retrograde trafficking, while it was expanded through inducing the lipid biosynthesis genes by the deletion of *OPI1* (Schuck et al. 2009).

In our previous study, we found that moderate overexpression of *SEC16* causes a lower specific growth rate and final biomass yield compared with the reference strain (Bao et al. 2017). Here, the retrograde trafficking engineered strains, YIGCS1 and YIGLO3, showed higher specific growth rate and higher final biomass yield, which indicated that cellular stress caused by *SEC16* overexpression was reduced by anaplerosis of the retrograde trafficking. This could be the result that the trafficking flows were balanced between the ER and the Golgi apparatus through engineering the retrograde trafficking in the anterograde trafficking amplifying strain. Accordingly, there was no indication of increased oxidative stress in strains YIGCS1 and YIGLO3, as measured by ROS, despite a higher flux through the secretory pathway. In addition, we also observed that the titers were increased and the biomass was slightly, but significantly, in the strains with a balanced trafficking compared with the only anterograde trafficking amplifying strain, when overexpressing two other recombinant proteins. This implied that this vesicle trafficking balanced system could be used a general strategy for designing improved recombinant protein producing strains.

In conclusion, our strategy may be generally applicable for improving recombinant protein production in yeast, as we have shown that the strategy resulted in improved secretion of three different recombinant proteins when *GLO3* was overexpressed. In conclusion, we report the positive effect of engineering the retrograde trafficking in a *SEC16* moderate overexpression yeast strain on the secretion of three different recombinant proteins. We detected increased ER membrane surface and an unchanged ROS accumulation in the engineered strains, which suggest that increased trafficking turnover benefits recombinant protein secretion as well as the cellular stress level.

## Additional file

**Additional file 1.** Additional table and figures.

## Abbreviations

ER: endoplasmic reticulum
UPR: unfolded protein response
ERES: endoplasmic reticulum exit site
COPII: coat protein complex II
COPI: coat protein complex I
ArfGEF: Arf1p guanine exchange factor
GAP: GTP activating protein
ROS: reactive oxygen species

## References

[CR1] Aridor M, Bannykh SI, Rowe T, Balch WE (1995). Sequential coupling between COPII and COPI vesicle coats in endoplasmic-reticulum to Golgi transport. J Cell Biol.

[CR2] Bao J, Huang M, Petranovic D, Nielsen J (2017). Moderate expression of *SEC16* increases protein secretion by *Saccharomyces cerevisiae*. Appl Environ Microbiol.

[CR3] Brandizzi F, Barlowe C (2013). Organization of the ER–Golgi interface for membrane traffic control. Nat Rev Mol Cell Biol.

[CR4] Carlson M, Taussig R, Kustu S, Botstein D (1983). The secreted form of invertase in *Saccharomyces cerevisiae* is synthesized from mRNA encoding a signal sequence. Mol Cell Biol.

[CR5] Cox J, Mann M (2011). Quantitative, high-resolution proteomics for data-driven systems biology. Annu Rev Biochem.

[CR6] de Ruijter JC, Koskela EV, Frey AD (2016). Enhancing antibody folding and secretion by tailoring the *Saccharomyces cerevisiae* endoplasmic reticulum. Microb Cell Fact.

[CR7] Echevarria W, Leite MF, Guerra MT, Zipfel WR, Nathanson MH (2003). Regulation of calcium signals in the nucleus by a nucleoplasmic reticulum. Nat Cell Biol.

[CR8] Feizi A, Österlund T, Petranovic D, Bordel S, Nielsen J (2013). Genome-scale modeling of the protein secretory machinery in yeast. PLoS ONE.

[CR9] Hawkins RD, Hon GC, Ren B (2010). Next-generation genomics: an integrative approach. Nat Rev Genet.

[CR10] Hong KK, Nielsen J (2012). Metabolic engineering of *Saccharomyces cerevisiae*: a key cell factory platform for future biorefineries. Cell Mol Life Sci CMLS.

[CR11] Hou J, Tyo K, Liu Z, Petranovic D, Nielsen J (2012). Engineering of vesicle trafficking improves heterologous protein secretion in *Saccharomyces cerevisiae*. Metab Eng.

[CR12] Hou J, Tyo KE, Liu Z, Petranovic D, Nielsen J (2012). Metabolic engineering of recombinant protein secretion by *Saccharomyces cerevisiae*. FEMS Yeast Res.

[CR13] Huang M, Bao J, Nielsen J (2014). Biopharmaceutical protein production by *Saccharomyces cerevisiae*: current state and future prospects. Pharm Bioprocess.

[CR14] Huang M, Bai Y, Sjostrom SL, Hallstrom BM, Liu Z, Petranovic D, Uhlen M, Joensson HN, Andersson-Svahn H, Nielsen J (2015). Microfluidic screening and whole-genome sequencing identifies mutations associated with improved protein secretion by yeast. Proc Natl Acad Sci USA.

[CR15] Idiris A, Tohda H, Kumagai H, Takegawa K (2010). Engineering of protein secretion in yeast: strategies and impact on protein production. Appl Microbiol Biotechnol.

[CR16] Jensen D, Schekman R (2011). COPII-mediated vesicle formation at a glance. J Cell Sci.

[CR17] Komano H, Rockwell N, Wang GT, Krafft GA, Fuller RS (1999). Purification and characterization of the yeast glycosylphosphatidylinositol-anchored, monobasic-specific aspartyl protease yapsin 2 (Mkc7p). J Biol Chem.

[CR18] Liu Z, Tyo KE, Martinez JL, Petranovic D, Nielsen J (2012). Different expression systems for production of recombinant proteins in *Saccharomyces cerevisiae*. Biotechnol Bioeng.

[CR19] Martínez JL, Meza E, Petranovic D, Nielsen J (2016). The impact of respiration and oxidative stress response on recombinant α-amylase production by *Saccharomyces cerevisiae*. Metab Eng Commun.

[CR20] Nielsen J (2013). Production of biopharmaceutical proteins by yeast: advances through metabolic engineering. Bioengineered.

[CR21] Pelham HRB (1995). Sorting and retrieval between the endoplasmic-reticulum and Golgi-apparatus. Curr Opin Cell Biol.

[CR22] Pepperkok R, Lowe M, Burke B, Kreis TE (1998). Three distinct steps in transport of vesicular stomatitis virus glycoprotein from the ER to the cell surface in vivo with differential sensitivities to GTP gamma S. J Cell Sci.

[CR23] Plath K, Mothes W, Wilkinson BM, Stirling CJ, Rapoport TA (1998). Signal sequence recognition in posttranslational protein transport across the yeast ER membrane. Cell.

[CR24] Poon PP, Cassel D, Spang A, Rotman M, Pick E, Singer RA, Johnston GC (1999). Retrograde transport from the yeast Golgi is mediated by two ARF GAP proteins with overlapping function. EMBO J.

[CR25] Reaves ML, Rabinowitz JD (2011). Metabolomics in systems microbiology. Curr Opin Biotechnol.

[CR26] Robinson AS, Hines V, Wittrup KD (1994). Protein disulfide isomerase overexpression increases secretion of foreign proteins in *Saccharomyces cerevisiae*. Bio/Technology.

[CR27] Schröder M (2008). Engineering eukaryotic protein factories. Biotechnol Lett.

[CR28] Schuck S, Prinz WA, Thorn KS, Voss C, Walter P (2009). Membrane expansion alleviates endoplasmic reticulum stress independently of the unfolded protein response. J Cell Biol.

[CR29] Shusta EV, Raines RT, Pluckthun A, Wittrup KD (1998). Increasing the secretory capacity of *Saccharomyces cerevisiae* for production of single-chain antibody fragments. Nat Biotechnol.

[CR30] Snyder M, Gallagher JE (2009). Systems biology from a yeast omics perspective. FEBS Lett.

[CR31] Solis-Escalante D, Kuijpers NG, Bongaerts N, Bolat I, Bosman L, Pronk JT, Daran JM, Daran-Lapujade P (2013). amdSYM, a new dominant recyclable marker cassette for *Saccharomyces cerevisiae*. FEMS Yeast Res.

[CR32] Supek F, Madden DT, Hamamoto S, Orci L, Schekman R (2002). Sec16p potentiates the action of COPII proteins to bud transport vesicles. J Cell Biol.

[CR33] Szul T, Sztul E (2011). COPII and COPI traffic at the ER–Golgi interface. Physiology.

[CR34] Tu BP, Weissman JS (2002). The FAD- and O(2)-dependent reaction cycle of Ero1-mediated oxidative protein folding in the endoplasmic reticulum. Mol Cell.

[CR35] Tyo KE, Liu Z, Petranovic D, Nielsen J (2012). Imbalance of heterologous protein folding and disulfide bond formation rates yields runaway oxidative stress. BMC Biol.

[CR36] Valkonen M, Penttila M, Saloheimo M (2003). Effects of inactivation and constitutive expression of the unfolded-protein response pathway on protein production in the yeast *Saccharomyces cerevisiae*. Appl Environ Microbiol.

[CR37] Verduyn C, Postma E, Scheffers WA, Van Dijken JP (1992). Effect of benzoic acid on metabolic fluxes in yeasts: a continuous-culture study on the regulation of respiration and alcoholic fermentation. Yeast.

[CR38] Ward TH, Polishchuk RS, Caplan S, Hirschberg K, Lippincott-Schwartz J (2001). Maintenance of Golgi structure and function depends on the integrity of ER export. J Cell Biol.

[CR39] Wittrup KD, Benig V (1994). Optimization of amino-acid supplements for heterologous protein secretion in *Saccharomyces cerevisiae*. Biotechnol Tech.

