# Supplementary material for: Balanced trafficking between the ER and the Golgi apparatus increases protein secretion in yeast
Source: AMB Express. 2018 Mar 12;8:37. doi: 10.1186/s13568-018-0571-x (PMC5847638; doi:10.1186/s13568-018-0571-x)
Supplement: Supplementary file 1 — Additional file 1. Additional table and figures. [file 13568_2018_571_MOESM1_ESM.pdf]

**AMB Express**

# Balanced trafficking between the ER and the Golgi apparatus increases protein secretion in yeast

*Jichen Bao[1,2], Mingtao Huang[1,2], Dina Petranovic[1,2] and Jens Nielsen[1,2,3,#]*

[1]Department of Biology and Biological Engineering, Chalmers University of Technology, SE41296 Gothenburg, Sweden
[2]Novo Nordisk Foundation Center for Biosustainability, Chalmers University of Technology, SE41296 Gothenburg, Sweden
[3]Novo Nordisk Foundation Center for Biosustainability, Technical University of Denmark, DK2970 Hørsholm, Denmark

[#]To whom correspondence should be addressed.
Email: nielsenj@chalmers.se
Tel: +46 31 772 38 04
Fax: +46 31 772 38 01

Table S1 Primers used in this study.

| Name | Sequence (5’ to 3’) |
|---|---|
| GCS1-up-100-F | gaaccaaattgcacaactatatcaagaatg |
| GCS1-up-100-R-amds | ctgcgcacgtcaagactgtcaaggagggtattctgggcctccatgtcgctctataatccgcgataaaattgc |
| amdS-F | gacatggaggcccagaatac |
| REC-amdSYM-R(gcs1) | atatagttgtgcaatttggttcttgacaaaaactggcgttgcttgcgggtaaccgcgcgacagtatagcgaccagcattcacatac |
| REC-P-TEF1-F(GCS1) | tcgcgcggttacccgcaagcaacgccagtttttgtcaagaaccaaattgcacaactatatatagcttcaaaatgtttctactcctttttttactcttc |
| gcs1-TEF1-R | gcaaaagacgcctgcgggtatctgggtccactttccaatctgacattttgttaaacttagattagattgctatgctttctttctaatgagc |
| GCS1-F | atgtcagattggaaagtggacccagatacccg |
| GCS1-MID-R | ccttttgaaaattggcaccattactag |
| GLO3-up-120-F | cgtaaacacgatttagctggc |
| GLO3-up-120-R-amds | ctgcgcacgtcaagactgtcaaggagggtattctgggcctccatgtctgtcgctatgtgtatactaaattgctc |
| REC-amdSYM-R(glo3) | aagaatagttgttttctttttttttttttttttcgaaactttaccgtgtcgtcgaaaatcagtatagcgaccagcattcacatac |
| REC-P-TEF1-F(glo3) | attttcgacgacacggtaaagtttcgaaaaaaaaaaaaaaaaaagaaaacaactattcttatagcttcaaaatgtttctactcctttttttactcttc |
| glo3-TEF1-R | gagtagtctgttccgtggcaaatgtttctccttcatcgttactcatTTTGTTaaacttagattagattgctatgctttctttctaatgagc |
| GLO3-F | atgagtaacgatgaaggagaaacatttgccacggaacagactactc |
| GLO3-MID-R | cttgaccgttcaagactaattctg |

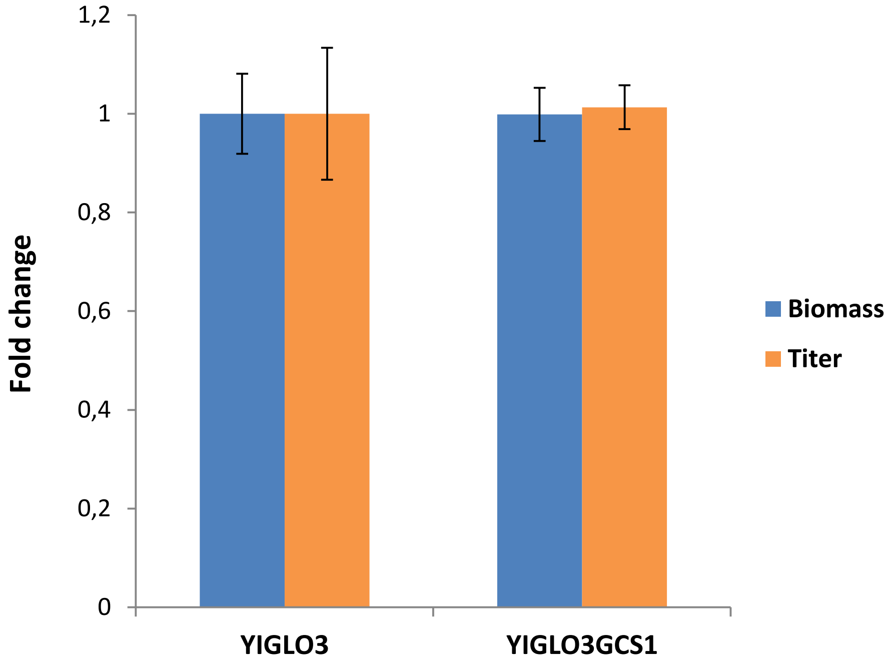


Fig. S1 Co-overexpression of *GCS1* and *GLO3* in YIGS16 does not further increase the protein secretion. Measurements are reported as the average value ± standard deviation from independent quintuplicates.

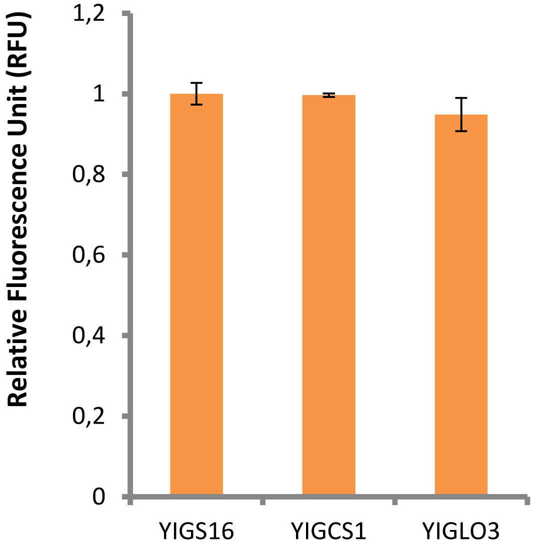


Fig. S2 Intracellular ROS levels of strains YIGS16, YIGCS1 and YIGLO3 using dihydrorhodamine 123. Measurements are reported as the average value ± standard deviation from independent triplicates.

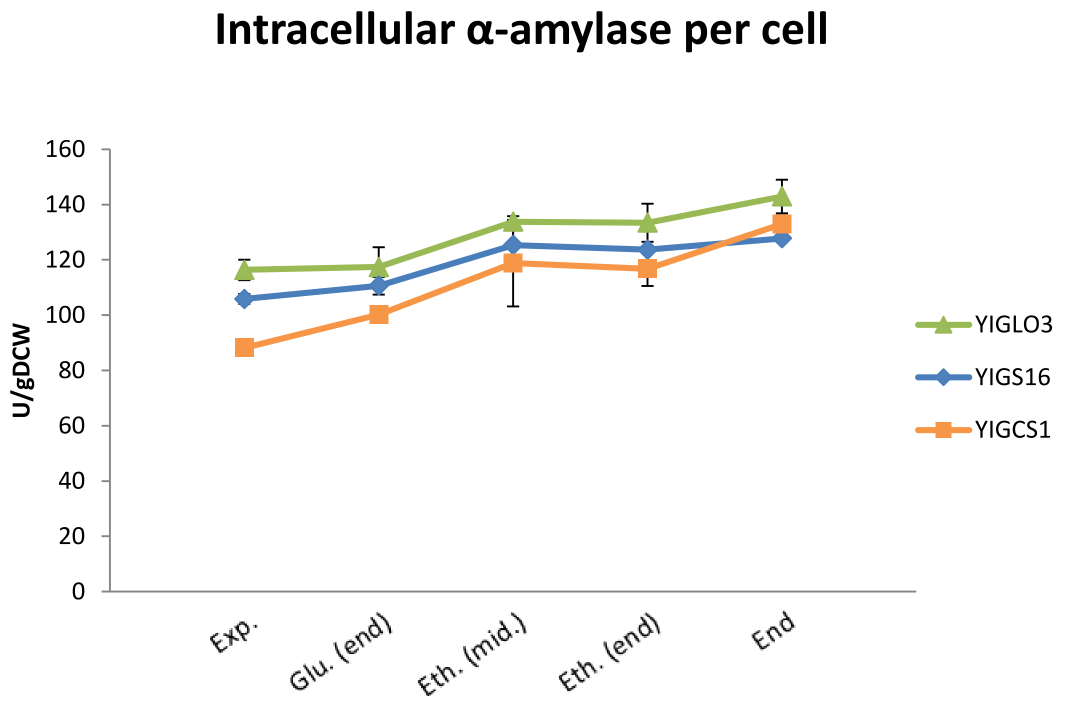


Fig. S3 The intracellular α-amylase per cell of the three strains. Measurements are reported as the average value ± standard deviation from independent triplicates.

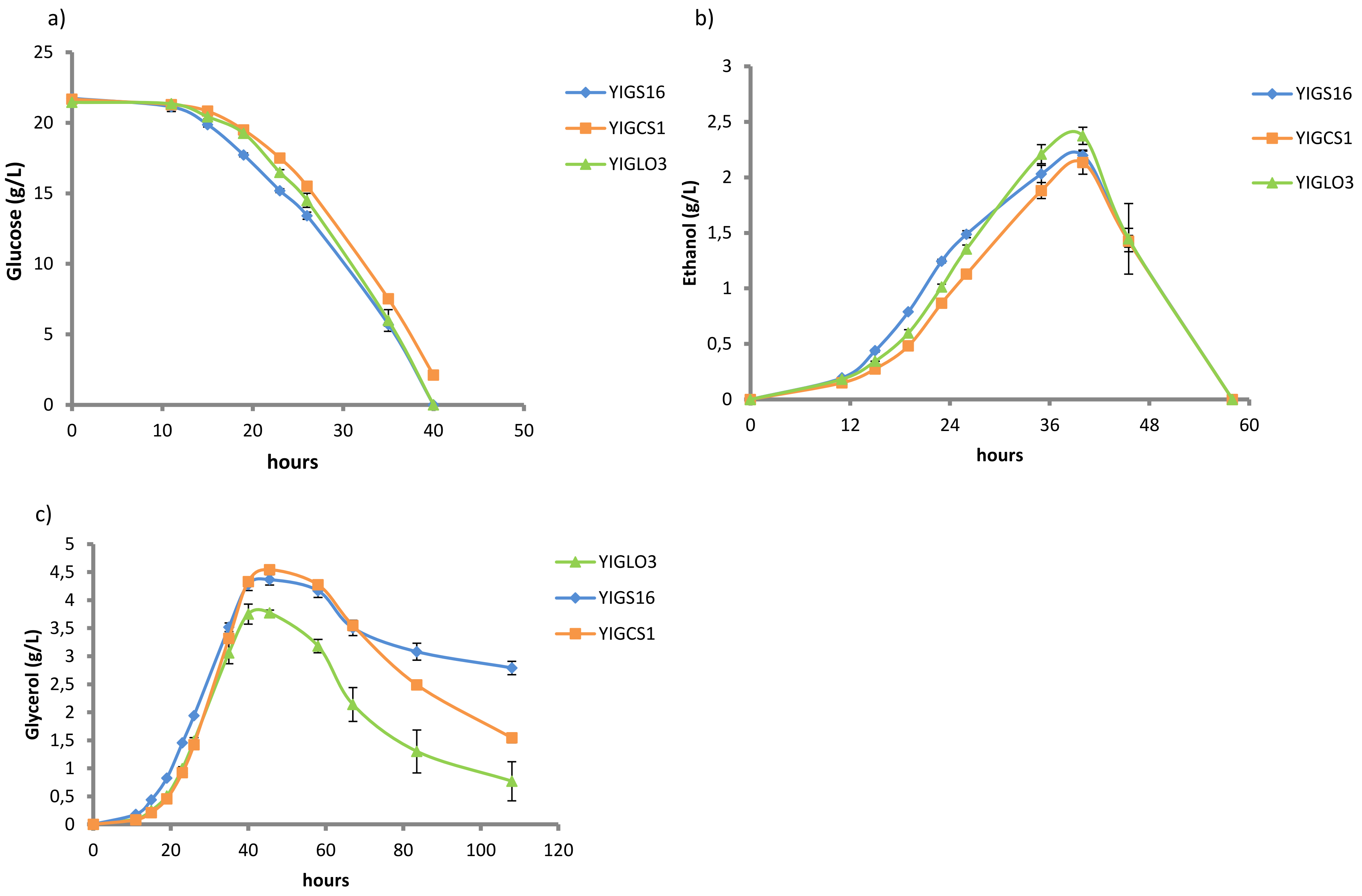


Fig. S4 a) the glucose consumption curves, b) the ethanol curves and c) the glycerol curves of the three strains. Measurements are reported as the average value ± standard deviation from independent triplicates.
